# Properties of a New Food Supplement Containing *Actinia equina* Extract

**DOI:** 10.3390/antiox9100945

**Published:** 2020-10-01

**Authors:** Marika Lanza, Giovanna Casili, Giovanna Loredana La Torre, Daniele Giuffrida, Archimede Rotondo, Emanuela Esposito, Alessio Ardizzone, Rossana Rando, Giovanni Bartolomeo, Ambrogina Albergamo, Rossella Vadalà, Andrea Salvo

**Affiliations:** 1 Department of Chemical, Biological, Pharmaceutical and Environmental Sciences, University of Messina, Viale Ferdinando Stagno D’Alcontres 3, 98166 Messina, Italy; mlanza@unime.it (M.L.); gcasili@unime.it (G.C.); aleardizzone@unime.it (A.A.); 2 Department of Biomedical and Dental Sciences and Morphofunctional Imaging, University of Messina, Polo Universitario Annunziata, 98168 Messina, Italy; llatorre@unime.it (G.L.L.T.); arotondo@unime.it (A.R.); rrando@unime.it (R.R.); gbartolomeo@unime.it (G.B.); aalbergamo@unime.it (A.A.); rosvadala@tiscali.it (R.V.); 3 Department of Chemistry and Drug Technology, University of Roma La Sapienza, via P.le A. Moro 5, 00185 Roma, Italy; andrea.salvo@uniroma1.it

**Keywords:** *Actinia equina*, food supplement, marine resources, antioxidant, anti-inflammatory

## Abstract

Marine species represent a great source of biologically active substances; *Actinia equina* (AE), an *Anthozoa Cnidaria* belonging to the *Actinidiae* family, have been proposed as original food and have already been included in several cooking recipes in local Mediterranean shores, and endowed with excellent nutraceutical potential. The aim of this study was to investigate some unexplored features of AE, through analytical screening and an in-vitro and in-vivo model. An in-vitro study, made on RAW 264.7 stimulated with H_2_O_2_, showed that the pre-treatment with AE exerted an antioxidant action, reducing lipid peroxidation and up-regulating antioxidant enzymes. On the other hand, the in-vivo study over murine model demonstrated that the administration of AE extracts is able to reduce the carrageenan (CAR)-induced paw edema. Furthermore, the histological damage due to the neutrophil infiltration is prevented, and this highlights precious anti-inflammatory features of the interesting food-stuff. Moreover, it was assessed that AE extract modulated nuclear factor kappa-light-chain-enhancer of activated B cells (NF-kB) and The nuclear factor erythroid 2–related factor 2 (Nrf-2) pathways. In conclusion, our data demonstrated that thanks to the antioxidant and anti-inflammatory properties, AE extract could be used as a new food supplement for inflammatory pathology prevention.

## 1. Introduction

The *Actinidiae* family comprises seven genera with ten species; these are solitary polyps, often devoid of skeletal structure and with the ability to attach themselves to the rocks of the seabed through their pedal disk. *Actinia equina* (AE), among *Actinidiae* species, is an intertidal, versatile polyp, easily spotted as a dark cover of rocks in shallow waters. AE has a wide array of colour variation, from green to red. The most common hue is rust-red. Anatomically, it is divided into three regions: the tentacles, the body cylinder (which houses the gastrovascular cavity, the pharynx, the gonads, and the retractor muscles), and the mentioned pedal disk. AE, and all anemones, show the beautiful light-coloured tentacles used to trap and ingest their prey. Cnidoblasts are storage cells housing the nematocyst (stinging organelles) which appear as blue spots at the end of the body column and inside the tentacles [1]. The crown of tentacles (up to 192) is arranged radially in six circles around the opening to the gastrovascular cavity [2]. Colours depend on the environmental conditions and general health. Two cell layers, with a thin acellular mesoglea in the middle make up the body; the inner cavity is called coelenteron, which has a digestive function, acting as both mouth and anus. The tentacles contain a strongly stinging poison that is used by the anemone to snatch small fish or crustaceans that make up its diet [1]. After all, the people along the Mediterranean shores have appreciated this sea gift as cooked dish since millennia: the toxins are thermally unstable, whereas the flavour keeps intact and strong after heating. AE, commonly called “sea tomato”, is frequently found in the Mediterranean Sea [3]; it is traditionally considered a delicious food yielding popular dishes in South Italy (Sardinia, Sicily, Puglia). Although it is not among the conventional marine animal species of the markets such as fish, molluscs and crustaceans, it is also right to take into account the edible value of the AE, which, moreover, and even though not in large quantities, are sought as a valuable resource [4]. AE is not a protected species; it reproduces easily due to being almost infesting, and this is why it can be used as a new functional food in the Mediterranean diet offering many mineral salts and nutrients such as carotenoids, fatty acids and sterols [5,6,7]. The purpose of this work was to evaluate the antioxidant and anti-inflammatory properties of AE by using an in-vitro model of oxidative stress on murine RAW 264.7 macrophage cell line and via in-vivo carrageenan (CAR)-induced paw edema model in rats, thus inducing acute inflammation. The overall chemical profile was also explored through different analytical means.

## 2. Materials and Methods

### 2.1. Chemicals

The solvents used in extractions and sample preparation were of analytical, HPLC (high performance liquid chromatography) or LC/MS (liquid chromatography coupled with mass spectroscopy) grade and were purchased from Sigma-Aldrich (Milan, Italy). Sterols and triperpenic dialcohol standards, deuterated water (D_2_O), TSP (3-(Trimethylsilyl) propionic-2,2,3,3-d_4_ acid sodium salt), 1 M PBS (phosphate buffer solution), NaN_3,_ 2,7-dichlorofluorescein and Sylon HTP [1,1,1,3,3,3-Hexamethyldisilane+Trimethylchlorosilane+Pyridine, 3:1:9], were obtained from Sigma-Aldrich (Milan, Italy). Formic acid (95–97%), single polyphenol standards, and fatty acid methyl ester (FAMEs) reference standards (C4–C24) were purchased from Supelco (Bellefonte, PA, USA). Carotenoid standards, namely astaxanthin, lutein, α-carotene, β-carotene and violerythrin were purchased from Extrasynthese (Genay, France). Protamex, Flavourzyme 500 MG, Neutrase 0.8 L, and Alcalase 2.4 L were obtained from Novozymes China Inc. (Guangzhou, China); the composition of commercial proteases was 1.1:1.0:0.9 for Protamex: Flavourzyme 500 MG: Alcalase 2.4 L, respectively.

For the FAMEs profiling, helium (99.9995% purity) was provided by Rivoira S.p.A. (Milan, Italy); *n*-heptane was purchased from PanReac AppliChem (Barcelona, Spain). PTFE (poly-tetra-fluoro-ethylene) syringe filters (0.45 μm and 0.20 μm) were from Gelman Sciences Inc. (Ann Arbor, MI, USA).

Except for specific products indicated, all compounds were purchased from Sigma-Aldrich (St. Louis, MO, USA). All other chemicals were of the highest commercial grade available. All stock solutions were prepared in non-pyrogenic saline (0.9% NaCl, Baxter, Milan, Italy). Primary antibodies, used for western blot analyses, were purchased from Santa Cruz Biotechnology (Dallas, TX, USA); nuclear factor of kappa light polypeptide gene enhancer in B-cells inhibitor alpha (anti-IkB-α) (#sc1643), nuclear factor kappa-light-chain-enhancer of activated B cells (anti-NF-kB) (#sc8008), Cyclooxygenase 2 (anti-Cox2) (sc-1746), inducible Nitric Oxide Synthase (iNOS) (anti-iNOS (#sc8310), Manganese superoxide dismutase (Mn-SOD) anti-Mn-SOD (#sc137254), and nuclear factor erythroid 2-related factor 2 (anti-Nrf2) (#sc365949). Secondary antibodies were purchased from Jackson ImmunoResearch.

### 2.2. Samples

**Figure 1:**
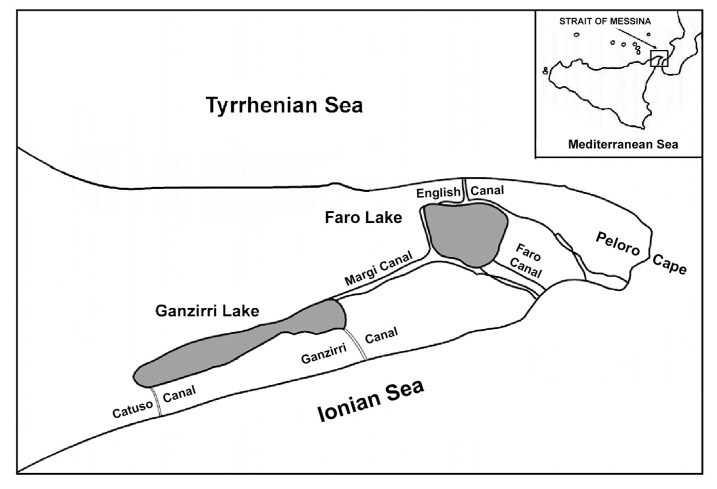
Representation of the Strait of Messina, place where *Actinia equina* (AE) specimens were collected.

The sampling of 1 kg specimens of AE took place in the Strait of Messina (Figure 1) near the breakwaters at a depth of about 1.5 m (GPS coordinates: 38°15′28.7″ N 15°37′12.9″ E). After collection, the samples were immediately brought with sea water to the laboratories where they were washed with fresh water, cleaned of stones and weighed.

Subsequently, the specimens were homogenized with a mixer, placed in a Becker and then in a freezer at −80 °C; the frozen sample was placed in an Alfa 2-4 LD freeze dryer plus CHRIST (Christ, Osterode am Harz, Germany) for two days until total loss of water. The dry sample was blended to make it poor and sieved to eliminate residues; it was weighed to calculate the water loss which was equal to 70%.

### 2.3. Lipid Extraction and Determination of Fatty Acids

Approximately 100 g of homogenized dry sample were placed in a pyrex glass bottle and 200 mL of *n*-hexane were added. The resulting mixture was stirred at room temperature on a plate with magnetic pawl for one hour and then placed in an ultrasonic bath thermostated at 25 °C for 30 min; the liquid phase was filtered with a 45 µm filter and dried with a Büchi Rotavapor R-215 rotavapor (Büchi, Milan, Italy). The yield in total lipids was 10%. 

Fatty acids methyl esters (FAMEs) were obtained from total lipids according to the procedure described by the EU Regulation 1833/2015 for olive oil. It consists of the hydrolysis of triacylglycerides and cold transesterification with a methanol KOH solution. Specifically, the methyl esters were prepared by vigorously shaking the solution of the oil in *n*-heptane (0.1 g in 2 mL) with 0.2 mL of the methanolic KOH solution for 30 s at room temperature (20 °C). After complete phase separation, the upper layer resulting solution was immediately injected into a gas chromatograph DANI MASTER GC-FID (Dany, Rome, Italy), equipped with a fused silica capillary column Phenomenex Zebron ZBWAX (Phenomenex, Bologna, Italy) (polar phase in polyethylene glycol) with a length of 30 m, internal diameter of 0.25 mm and film thickness of 0.25 μm. Helium was used as a carrier gas at a column flow rate of 1.2 mL/min, with a split ratio of 1:100. The temperature of the injector (split/splitless) and detector was of 220 °C and 240 °C, respectively. The oven was programmed as follows: initial temperature at 130 °C, final temperature at 200 °C (10 min) with an increase of 3 °C/min. The fatty acid methyl esters were identified by comparing the retention times with those of standard compounds. The relative percentage area of the fatty acids was obtained using the following relationship: %FAX = [AX/AT] × 100, where FAX stands for fatty acids to quantify, AX is the area of the methyl-esters and AT is the total area of the identified peaks in the chromatogram.

### 2.4. Phytosterols and Triterpenic Alcohols Analysis

The oils extraction for analysis of phytosterols and triterpenic dialcohols was conducted according to the official method reported in European Union Regulation 1348/2013 [8]. The lipid fraction samples were submitted to saponification with an ethanol potassium hydroxide solution, previous addition of an internal standard (5α-cholestane-3β-ol) for phytosterols quantification [9]. After the evaporation of the ethanol under vacuum, the unsaponifiable matter was extracted with diethyl ether and treated on a silica gel plate chromatography to separate the fraction constituted by phytosterols and triterpenic dialcohols. This fraction was derivatized with Sylon HTP to obtain the corresponding trimethylsilylethers (TMSEs) and subsequently analysed by gas chromatography, using a chromatograph DANI MASTER GC (Dany, Rome, Italy), equipped with a capillary column (ZB-1 Phenomenex: 15 m × 0.25 mm, 0.25 μm film thickness, (Phenomenex, Bologna, Italy)) and a flame ionization detector (Dany, Rome, Italy). The injector was operated in split mode (ratio 1:50) and the injection volume was 1 μL. The operating conditions were as follows: carrier gas: helium at 1 mL/min; injector and detector temperature: 290 °C; column temperature was programmed to 240 °C for 5 min and then ramped up at 2 °C/min to 290 °C for 5 min. The phytosterols and triterpenic dialcohols were identified by using commercial standards. According to EU Regulation 1348/2013 [8], the apparent β-sitosterol was calculated as the sum of Δ-5,23-stigmastadienol, cholesterol, β-sitosterol, sitostanol, Δ-5-avenasterol and Δ-5,24-stigmastadienol. The relative amount of each sterol and triterpenic dialcohol was expressed as a percentage of total sterols, while the total sterols were expressed as the sum of single phytosterols (mg/kg).

### 2.5. Carotenoids Extraction and Analysis by High Performance Liquid Chromatography Coupled to a Diode Array Detector with Atmospheric Pressure Chemical Ionization and Mass Spectrometry (HPLC-DAD-APCI-MS)

Carotenoids were extracted from AE lyophilized sample according to a method proposed by Salvo et al. [10] and all the treatments were performed under subdued light. To 0.5 g of lyophilized sample, 10 mL *n*-hexane were added and underwent five cycles of ultrasound-assisted extraction, lasting ten minutes each, at the controlled temperature of 25 °C. The resulting mixture was centrifuged for 10 min at 4000 rpm and at 25 °C. The upper layer was taken and dried by a Büchi Rotavapor R-215 (Büchi, Milan, Italy). The dry matter was dissolved in 10 mL of ethyl acetate to run the same procedure described for the *n*-hexane again. After the second evaporation, the residue was dissolved in 2 mL of methyl-terz-butyl-ether (MTBE)/methanol (1:1, *v/v*), filtered through 0.45 μm PTFE and analyzed by HPLC.

The analyses were carried out using an HPLC system (Shimadzu, Milan, Italy) equipped with a CBM-20A controller, two LC-20AD pumps, a DGU-20A3 degaser, a SIL-20AC auto-sampler and a SPD-M20A photo-diode array detector. The data were processed with the software Lab solution ver. 5.10.153 (Shimadzu, Milan, Italy). For MS analyses was used a mass spectrometer detector (LCMS-2020, Shimadzu, Milan, Italy), equipped with an APCI interface, both in positive and negative ionization mode. Separations were performed on a C30 YMC column (250 × 4.6 mm; 5 μm) and the injection volume was 20 μL. The mobile phase consisted of a binary gradient of methanol/methyl-terz-butyl ether/water (MeOH/MTBE/H_2_O) (90:8:2; *v/v/v*) (A), and MeOH/MTBE/H_2_O (8:90:2; *v/v/v*) (B), starting with 0% B, and then a linear gradient increase to 30% of B in 20 min, and to 80% B at 35 min, and to 100% B at 65 min and to 100% B at 75 min, then re-equilibrating the column to initial B concentration at a flow rate of 1 mL/min. Pigments in the sample were identified by comparison with available standards, elution order, UV/vis (ultra violet and visible) spectra, their APCI-MS spectra, recorded both in positive and negative ionization modes, and where available, by literature information. The UV–Vis spectra were acquired in the range of 250–600 nm, while the chromatograms were extracted at 450 nm (sampling frequency: 1.5625 Hz; time constant: 0.64 s). The MS parameters were set as follows: Acquisition Mode, Scan; Interface Temperature, 350 °C; Interface Voltage: 4.5 kV; Heat Block, 300 °C; CDL Voltage: 0 V; CDL temperature, 300 °C; *m/z* range, 300–1200; Nebulizing gas flow (N_2_): 4 mL/min; Event Time: 1 s; Detector Voltage: 0.8 kV; Q-array: 0.0 V; RF: 90 V; Sampling: 2 Hz. Quantification by HPLC was performed with the external standard method, for available standards; standard curves were calculated by the linear regression analysis and any quantification was estimated as mean value of three repeated measurements.

### 2.6. Polyphenols Extraction and Determination by Ultra High Performance Liquid Chromatography Coupled to a Diode Array Detector with Atmospheric Pressure Chemical Ionization and Mass Spectrometry (UHPLC-DAD-MS) 

The polyphenols were extracted from a dry pulverized sample (5 g) with 50 mL of methanol. The resulting mixture was vortexed for 1 min, placed in ultrasonic bath for 30 min at the controlled temperature of 35 °C and then centrifuged for 25 min at 5000 rpm at room temperature. The organic layer was then separated, and the resulting material was treated with an additional 50 mL of methanol. After vortex and ultrasonic treatment, centrifugation, removal of the methanol layer and union to the original organic phase removed, the process was repeated a third time. The combined methanol extracts were evaporated to dryness, at 25 °C under reduced pressure, by a Büchi Rotavapor R-215. The dried sample was then dissolved in 2 mL of water/acetonitrile (1:1, *v/v*), filtered through a 0.20 μm PTFE syringe filter and analyzed by reversed phase ultra-high-performance liquid chromatography coupled to diode-array detection and mass spectrometry (RP-UHPLC-DAD-MS) for the qualitative-quantitative determination of single polyphenols. Analyses were conducted by a Shimadzu Prominence UFLC XR system (Shimadzu, Milan, Italy), equipped with a CBM-20A controller, a LC-20AD-XR binary pump system, a DGU-20A3R degasser, a SPD-M20 detector, a CTO-20AC column oven and a SIL-20A-XR auto-sampler. The LC system was interfaced through an electrospray ionization (ESI) source to an LCMS-8040 triple quadrupole mass spectrometer (Shimadzu, Milan, Italy). Data collection and handling was performed by LabSolution software v. 5.53 (Shimadzu, Milan, Italy). Chromatographic separations occurred on an Ascentis Express C18 (250 × 4.6 mm I.D. × 2.7 μm d.p.) (Supelco, Bellefonte, Pennsylvania, USA); whereas mobile phases were constituted by water/formic acid (99.9:0.1, *v/v*) (solvent A), and acetonitrile/formic acid (99.9:0.1, *v/v*) (solvent B). It was assured that only ~1/3 of the total flow was directed from the LC system to the ESI-MS by means of a stainless-steel splitting device (VICI AG International, Schenkon, Switzerland).

The method of analysis was adapted from the protocol proposed by Certo et al. [11] and consisted of the following gradient program: 0 min, 5% B, 5 min, 5% B, 15 min, 30% B, 40 min, 60% B, 45 min, including also final column washing and re-equilibrating steps. The mobile phase flow rate was 1 mL/min, while the oven temperature and injection volume were, respectively, set at 30 °C and 5.0 μL. The DAD spectra were acquired in the range 190–400 nm, and the chromatograms were extracted operating at wavelengths between 280 nm and 370 nm (time constant: 0.60 s; sample frequency: 1.5625 Hz). MS acquisition was performed using ESI interface in negative mode, and operating in full-scan (*m/z* 100–800) and selected ion monitoring (SIM) modes according to the following conditions: interval, 1.0 s; scan speed, 715 amu/s; nebulizing gas (N_2_) flow, 1.5 L/min; drying gas (N_2_) flow, 10 L/min; ESI temperature, 350 °C; heat block, 300 °C; DL (desolvation line) temperature, 300 °C; DL voltage −34 V; probe voltage, +4.5 kV. Measurements were conducted in triplicate along with analytical blanks (methanol). 

The investigated compounds were: *p*-coumaric acid, ferulic acid, gallic acid, chlorogenic acid, caffeic acid, *p*-hydroxybenzoic acid, vanillic acid, methyl gallate, catechin, epicatechin, quercetin, rutin, luteolin, luteolin 7-glucoside, quercetin-3-glucoronide and kaempferol-3-glucoronide. Due to the absence of comparative literature reporting single polyphenols for AE, phenols were simply selected based on the standard availability and different chemical classes. An external calibration procedure was conducted for quantification purposes. In particular, five-point calibration curves were constructed in the range of 1–100 mg/L, by serially diluting a stock solution of each commercial standard (1000 mg/L) in methanol. All measurements were conducted in triplicate. Compound identification was based on calculated exact mass and retention time of each target compound.

### 2.7. Aminoacidic Extraction and NMR Analyses

Aminoacids determination was performed according to the protocol proposed by Mangano et al. [12]. Small aliquots of frozen AE were thawed, ground and mixed with distilled water with a weight-to-volume ratio of 1:3. The sample was kept in alkali (NaOH) for 3 h at 70 °C; this procedure leads to the total cleavage of the peptide bonds of any peptide or protein structure in AE samples. The matter was then filtered through a filter press and centrifuged at 8000 rpm for 15 min at 4 °C. Successively, the upper clear solution was collected and dried using a Mini Spray Dryer B-290 (Büchi, Milan, Italy). The resulting dry powder (200 mg) was later dissolved in 900 μL of water and 100 μL of a suitable solution for the NMR (Nuclear Magnetic Resonance) analysis.

According to the well assessed procedure [13,14], the final solution was made up of H_2_O/D_2_O with 1:9 volumetric ratio and with 1 mM of TSP (3-(Trimethylsilyl) propionic-2,2,3,3-d_4_ acid sodium salt, used as NMR frequency and quantification standard with δ = 0.0 ppm), 600 mM of phosphate buffer solution (to keep costant pH = 7.24), and 0.2% of NaN_3_ (able to inhibit interfering bacterial growth). The resulting solution was poured into a 5 mm NMR text-tube. The NMR analysis was carried out at 25 °C with a NMR Bruker Avance III 500 MHz spectrometer (Bruker, Milan, Italy) equipped with a SMARTprobe with gradients. After optimization of the field homogeneity and calculation of the 90° pulse (8 μs @ 3dB) the ^1^H-NMR *noesypresat* experiments were run to guarantee water suppression and detection and quantification of free α-amino acids according to the Multiple Assignment Recovered Analysis (MARA-NMR) quantification technique [13,14].

### 2.8. In-Vitro Study

#### 2.8.1. MTT Assay

Murine RAW 264.7 macrophage cell line was acquired from American Type Culture Collection (ATCC) and cultured as reported by [15]. The cellular viability of RAW 264.7 was assessed by MTT assay as previously described by [16]. The extent of reduction of MTT to formazan was quantified by 232 measurement of optical density at 550 nm (OD550) with a microplate reader (Multiskan FC Thermo Scientific, Waltham, MA, USA). In another set of experiments to test antioxidant properties of AE extract, cells were pre-treated with AE extract at different concentrations and were then stimulated with H_2_O_2_ at the final concentration of 200 μM for 10 min.

#### 2.8.2. Malondialdehyde Assay (MDA Assay)

MDA assay on RAW 264.7 (1 × 10^5^ cells/well) was performed as reported by [17].

#### 2.8.3. Western Blot Analysis

RAW 264.7 were washed twice with ice-cold phosphate-buffered saline (PBS), harvested and resuspended in Tris-HCl 20 mM pH 7.5, NaF 10 mM, 150 μL NaCl, 1% Nonidet P-40 and protease inhibitor cocktail (Roche, Basel, Swiss). After 40 min, cell lysates were centrifuged at 12,000 rpm for 15 min at 4 °C. Protein concentration was estimated by the Bio-Rad protein assay using bovine serum albumin as standard. The following primary antibodies were used: anti-manganese superoxide dismutase (Mn-SOD) (1:500; Santa Cruz Biotechnology, Dallas, TX, USA) and anti-heme oxygenase-1 (HO-1) (1:500; Santa Cruz, Dallas, TX, USA). The relative expression of the protein bands was quantified by densitometry with BIORAD ChemiDoc TMXRS + software (Hercules, CA, USA) and standardized to β-actin.

### 2.9. In Vivo Study

#### 2.9.1. Animals

The study was carried out on Male Wistar rats weighing 200–230 g (Envigo, Milan, Italy), were housed in a controlled environment (22 ± 2 °C, 55 ± 15% relative humidity, 12 h light/dark cycle). This study was approved by the University of Messina Review Board for the care of animals, in compliance with Italian regulations on protection of animals (n° 549/2018-PR released on 16/7/2018). Animal care was in accordance with Italian regulations on the use of animals for the experiment (D.M.116192) as well as with EEC regulations (O.J. of E.C. L 358/1 12/18/1986).

#### 2.9.2. CAR-Induced Paw Edema

Edema induction was performed by injecting CAR (100 μL of a 1% suspension in 0.85% saline) into the right paw of each rat [18]. The volume of paw edema and variations in paw volume were measured as described by [18]. 

#### 2.9.3. Experimental Groups

Rats were divided into the following groups with different doses of AE extract:

Group 1: Sham + vehicle: The sham-operated group received orally saline instead of CAR (*n* = 10).

Group 2: CAR + vehicle: rats were subjected to CAR-induced paw edema and received orally saline (*n* = 10);

Group 3: CAR + AE extract (10 mg/kg): rats were administered 30 min before CAR-induced paw edema with AE, 10 mg/kg, by oral gavage (*n* = 10);

Group 4: CAR + AE extract (30 mg/kg): rats were administered 30 min before CAR-induced paw edema with AE, 30 mg/kg, by oral gavage (*n* = 10);

Group 5: CAR + AE extract (100 mg/kg): rats were administered 30 min before CAR-induced paw edema with AE, 100 mg/kg, by oral gavage (*n* = 10).

At the end of the experiment, 6 h following CAR injection, animals were sacrificed under anesthesia [18], hind paws were removed and processed for biochemical analysis and histological examinations.

#### 2.9.4. Histological Examination

To evaluate the local inflammatory response, structural tissue changes of the muscle fibers and the activity of our extract, seven-micrometer-thick sections stained with hematoxylin and eosin (H&E) Bio-Optica, Milano, Italy). Histological estimations were made as previously described [19].

Each image was inspected with an optical microscope (Axostar Plus equipped with Axio-Cam MRc, Zeiss, Oberkochen, Germany). The histological results were shown at 10× (100 μm of the Bar scale). The degree of paw damage was evaluated according to a six-point score: 0 = no inflammation, 1 = mild inflammation, 2 = mild/moderate inflammation, 3 = moderate inflammation, 4 = moderate/severe inflammation and 5 = severe inflammation as reported by Petrosino et al. [20].

#### 2.9.5. Myeloperoxidase (MPO) Activity

MPO is an enzyme present in a particular type of white blood cell: polymorphonuclear leukocytes, mainly neutrophils and monocytes. This enzyme belongs, in particular, to the class of oxide-reductase, capable of detoxifying cells from the presence of reactive oxygen species, such as peroxides [21]. MPO activity was detected as previously described by Casili et al. [22]. 

#### 2.9.6. Western Blot Analysis

For nuclear factor of kappa light polypeptide gene enhancer in B-cells inhibitor alpha (IκB-α), nuclear factor kappa-light-chain-enhancer of activated B cells (NF-κB), Cyclooxygenase 2 (COX-2), inducible Nitric Oxide Synthase (iNOS), Manganese superoxide dismutase (Mn-SOD) and nuclear factor erythroid 2-related factor 2 (Nrf2).

Cytosolic and nuclear extracts of hind paw tissues were performed as previously described [23]. The levels of IκB-α, COX-2, iNOS and Mn-SOD were quantified in the cytosolic fraction, while NF-κB and Nrf-2 levels were quantified in the nuclear fraction.

Western Blots were made as previously described [24]. Filters were blocked with 1 × PBS, 5% (*w/v*) nonfat dried milk (PM) for 40 min at room temperature and then probed with one of the other primary antibodies: anti-IkB-α (1:500), anti-NF-kB (1:500), anti-Cox2 (1:500), anti-iNOS (1:500), anti-Mn-SOD (1:500), anti-Nrf2 (1:500) in 1 × PBS, 0.1% Tween-20, 5% *w/v* nonfat dried milk (PMT) at 4 °C, overnight. Membranes were incubated with peroxidase-conjugated bovine anti-mouse IgG secondary antibody or peroxidase-conjugated goat anti-rabbit IgG (1:2000, Jackson ImmunoResearch, West Grove, PA, USA) for 1 h at room temperature. Blots were also incubated with primary antibody against β-actin protein (1:10,000; Sigma-Aldrich Corp., St. Louis, USA) or laminin (1:10,000; Sigma-Aldrich Corp.), used as internal standards. Signals were detected with an enhanced chemiluminescence detection system reagent. Relative expression of protein bands was quantified by densitometry (optical density [OD] per mm^2^) with ChemiDoc™ XRS+ (Image Lab version 5.2.1 build 11, Bio-Rad Laboratories, Hercules, CA, USA). The relative expressions of the protein bands of IkB-α (37 kDa), NF-kB p65 (65 kDa), COX-2 (70 kDa), iNOS (131 kDa), MnSOD (25 kDa) and Nrf2 (65 kDa) were detected and quantified by densitometry as previously explained by Cordaro et al. [24]; densitometric analysis is related in each figure.

### 2.10. Statistical Analysis

All values are expressed as mean ± standard error of the mean (SEM) of N observations. Each analysis was performed three times with three samples replicates for each one. The results were analyzed by one-way ANOVA followed by a Bonferroni post-hoc test for multiple comparisons. A *p*-value of less than 0.05 was considered significant.

## 3. Results

### 3.1. Fatty Acids

Sea anemones have long been studied for the composition of fatty acids and the investigations have covered several *Actinaria* species [25,26,27,28,29]. In the present study, a total of 36 fatty acids were identified in AE and the proportions are reported in Table 1. Specifically, they were: 16 total saturated fatty acids (SFAs), 9 monounsaturated fatty acids (MUFAs) and 11 polyunsaturated fatty acids (PUFAs).

The fatty acid profile observed in the whole organism of AE indicates high percentage of total SFAs that accounted for 50.54%, while total MUFAs and total PUFAs total contents are almost comparable (23.26% and 26.20%, respectively). The main SFAs of AE were palmitic (C16:0) and stearic (C18:0) acids and this is in accordance with other precedent determinations for AE [27] and *Actinia tenebrosa* [28]. Very low content were detected for lauric (C12:0) acids, and this data is in accordance with the levels detected in AE in more recent time [30]. For the first time we reported the content of butyric (C4:0), caproic (C6:0), caprylic (C8:0), capric (C10:0), undecanoic (C11:0) and tricosylic (C23:0) acids that were detected at low content and for which we have not any reference data for comparison.

Among MUFAs, highest levels were detected for oleic (C18:1(n-9)) and vaccenic (C18:1(n-7)) acids, and these values together with le appreciable amount of palmitoleic (C16:1(n-7)) and *cis-11*-eicosenic (C20:1(n-9) acids are in accordance with precedent study by Stefanov et al. [27]. The differences observed for these MUFAs by Yatkin et al. [30] probably arise from differences in food chain in different sea ecosystems.

Lastly, appreciable amounts of PUFAs were determined and only stearidonic acid (C18:4 (n-3) was not detected. In particular, *cis*-*5,8,11,14,17*-eicosapentaenoic acid (C20:5(n-3)) was the most abundant, but significant levels of other acids were determined also and their contents followed the order: *cis-4,7,10,13,16,19*-docosahexaenoic acid (C22:6(n-3)) > arachidonic acid (C20:4(n-6)) > *cis-8,11,14*-eicosatrienoic acid (C20:3(n-6)) > linoleic acid (C18:2(n-6)) > *cis-11,14*-eicosadienoic acid (C20:2(n-6)). Almost all the data are in agreement with previous determinations on AE an some other actiniaria species [27,28,30]. Little amount variations could be related to species, seasonal change in chemical composition and different sea ecosystems.

The n-6/n-3 ratio was used to detect alteration due to feed consumption. A low value indicate that the total n-3 fatty acid content is high, as it should be for a marine organism, and the n-6 fatty acids are low, as they are scarce in marine environment. This is an important result for the possible consumption of AE as an aliment; in fact, several studies suggest that a diet with a low n-6/n-3 ratio can be associated with beneficial effects on cardiovascular or chronic disease [31]. 

### 3.2. Phytosterols and Triterpenic Alcohols

The sterol composition of AE was investigated for the first time in 1909 [32], whereupon initially only cholesterol was found. In the present investigation a total of 14 compounds have been characterised including cholesterol. To the best of our knowledge, this is the first work that reports the profile of specific phytosterols and triterpenic dialcohos in AE, since precedent investigation on green form of this marine animal evidenced only that in this species there was a decrease of the cholesterol concentration corresponding to an increase of the phytosterols. The compounds detected in the present investigation are reported in Table 2.

The obtained data evidenced once more that cholesterol is the predominant sterol, in accordance with other precedent studies on AE and other actiniaria species [7,33]. As the reported data show, of the 13 compounds investigated, only 11 have been identified since stigmasterol and sitostanol were below the detection limit. The concentrations of the identified compounds varied significantly and β-sitosterol was the most abundant. The second in order of importance was ergosterol, followed by Δ-5-avenasterol and campesterol. Unfortunately, due to the absence of comparative literature reporting single phytosterols and triterpenic dialcohos, we cannot compare our data with other ones. It could probably be assumed that the studied compounds, being secondary metabolites, are of phytoplankton origin or may be related to the common presence of macroalgal fragments in AE coelenterons. 

### 3.3. Carotenoids

It is well known that marine animals contain various carotenoids and since 1928 it was demonstrated polymorphism among individuals for different proportion of the carotenoids responsible for coloration of see anemones [6,34,35,36]. Among the 750 reported carotenoids, more than 250 originated from marine animals and algae, although it is now established that carotenoids in marine animals are directly accumulated from food or partly modified through metabolic reactions [6]. High performance liquid chromatography coupled to a diode array detector with atmospheric pressure chemical ionization and mass spectrometry (HPLC-DAD-APCI/MS) technique has proved very useful for the unambiguous determination of carotenoids in complex matrices. In this study, we report the characterization of carotenoids in unsaponified extracts of EA by HPLC-DAD-APCI/MS using a negative APCI ionization mode. The online use of HPLC with DAD and mass spectrometer detectors allowed for the easy identification of six carotenoids whose concentration is reported in Table 3.

The identified compounds were confirmed by comparing the chromatographic and spectral data with those of corresponding standards where possible. Additionally, identifications were supported considering the elution order and literature data. The present study confirmed the occurrence of astaxanthin, lutein, actinioerythrin, violerythrin, α- and β-carotenes which had been found earlier by nearly all investigations on carotenoids in this species [34,36,37,38]. Interestingly this study revealed that, contrary to reports of previous studies [34,36,38,39], violerythrin was the dominant carotenoid (1328.6 mg/kg), while the concentration of actionioerythrin (260.2 mg/kg), which is reported as the main carotenoid of AE in several papers, was one of the components present in the least amount, after lutein (150.2 mg/kg). The present study on AE confirmed the good occurrence of β- and α-carotene (550.6 mg/kg and 344.1 mg/kg, respectively) as reported in the first studies [34,37]. Concerning actinioerythrin small amounts, it may be considered that this compound was stated to be present as a protein complex [38], and so therefore the extraction condition can influence the final yield. Moreover, the possibility of using new and more efficient technologies, such as HPLC-DAD-MS, with the use of C30 columns, can be considered an excellent tool for unambiguous identification of carotenoids in complex matrix.

### 3.4. Polyphenols

The analysis of the unsaponified polyphenol extracts is challenging because of their complexity and it is an important aspect considering the beneficial health effects associated with the presence of polyphenols in food [40]. To the best of author’s knowledge, the polyphenol profile of AE has not yet been characterized elsewhere. Hence, a direct comparison of the present findings with previous literature data results is somehow arduous, due the absence of studies on polyphenols from this marine organism. As stated in the precedent section, due to the absence of comparative literature data reporting single polyphenols in AE, phenols were simply selected on the standard availability and different chemical classes. Of the sixteen compounds investigated (*p*-coumaric acid, ferulic acid, gallic acid, chlorogenic acid, caffeic acid, *p*-hydroxybenzoic acid, vanillic acid, methyl gallate, catechin, epicatechin, quercetin, rutin, luteolin, luteolin 7-glucoside, quercetin-3-glucoronide, and kaempferol-3-glucoronide), only five were identified. As reported in Table 4, most of the common phenolic acids were not detected with the exception of *p*-coumaric acid amounting to 8.03 mg/kg. However, some flavonoids, namely quercetin (51.12 mg/kg), rutin (36.22 mg/kg), catechin (15.32 mg/kg) and luteolin (2.53 mg/kg), were revealed.

While being an animal organism, AE demonstrated to be a discreet source of plant secondary metabolites. This may be related to the common presence of macroalgal fragments in its coelenteron content, which are probably not metabolized due to the absence of a suitable enzymatic digestion system [2,41].

### 3.5. Amino Acids

The evaluation of amino acids composition in AE was performed following a preliminary enzymatic approach on the matter and stabilising the resulting product by a smart low-cost spry drying procedure, as reported by Mangano et al. [12]. The amino acids composition of AE hydrolysate analysed by NMR is reported in Table 5.

The qualitative and quantitative determination was carried out by nuclear magnetic resonance (MNR), bypassing the usual pre- or post-column derivatization of each amino acid, as generally employed in HPLC methods. Conversely, the NMR analytical strategy, through minimal sample treatment and the use of a novel algorithm based on the multi assignment approach MARA-NMR [13], allows a quantification suitable for our purpose without the use of standard because the signal assignment for any amino acids in the prepared solution is well known. Moreover, the employ of NMR for analytical purpose was supported by the finding that there is an acceptable fitting between HPLC and NMR techniques [13].

The analytical procedure allowed the determination of 13 amino acids. The amino acids more representative into the hydrolyzed AE were in order: isoleucine (ILE), glutamic acid (GLU) and glycine (GLY). Leucine (LEU), lysine (LYS), valine (VAL), phenylalanine (PHE), arginine (ARG) and tyrosine (TYR) showed a comparable content while methionine was determined in lower amount.

Unfortunately, we cannot make any comparison of the actual finding with precedent literature data because, to the best of our knowledge, all the previous investigation on AE were reporting the amino acids sequence of specific proteins, nucleotides or protein related to specific gene structure.

### 3.6. Antioxidant Effect of AE Extract in RAW 264.7 Cell Cultures Stimulated with H_2_O_2_ and in MDA Level

**Figure 2:**
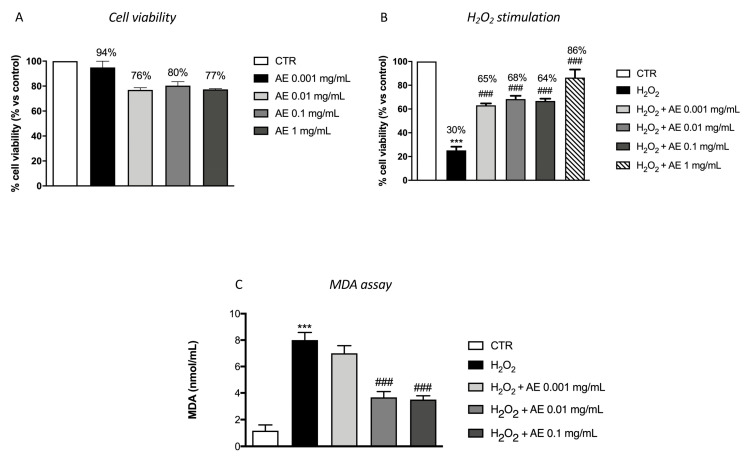
Antioxidant effect of AE extract in RAW 264.7 cultures stimulated with H_2_O_2_ and in MDA level. Cell viability test of AE extract (0.001 mg/mL, 0.01 mg/mL, 0.1 mg/mL, and 1 mg/mL) didn’t shown any significant cytotoxicity (**A**). Pre-treatment with AE extract at the concentration of 0.001 mg/mL, 0.01 mg/mL and 0.1 mg/mL and 1 mg/mL significantly prevented H_2_O_2_ cytotoxicity (**B**). MDA levels were significantly increased in the cell lysates following H_2_O_2_ stimulation. Lipid peroxidation was significantly attenuated by the pre-treatment with the extract at 0.01 and 0.1 mg/mL (**C**). Data are representative of at least three independent experiments; One-Way ANOVA test. *** *p* < 0.001 vs. Ctr; ### *p* < 0.001 vs. H_2_O_2_.

To evaluate the effect of AE extract on cell viability, RAW 264.7 cells were incubated with increasing concentrations of AE extract: 0.001, 0.01, 0.1 and 1 mg/mL. After 24 h of incubation, AE extract at the different concentrations showed viability almost comparable to the control group at all concentrations (Figure 2A). So, we examined the antioxidant effect of pre-treatment with AE-extract on Raw 264.7 cells stimulated with H_2_O_2_ (200 μM) for 10 min. As showed, Raw 264.7 cells stimulated with H_2_O_2_ reduced cells viability and translated in a decrease of MTT metabolism. Pre-treatment with different concentrations of AE extract, 2 h before of H_2_O_2_ stimulation, reduced cell death compared to the H_2_O_2_ group at the four concentrations (Figure 2B). Membrane phospholipids are susceptible to the attack of free radicals during oxidative stress. For this reason, we evaluated by MDA assay the lipidic peroxidation of the membrane. Our data showed a significant increase of MDA level after H_2_O_2_ stimulation (Figure 2C) while pre-treatment with AE extract at the concentration of 0.01 and 0.1 mg/mL significantly attenuated the levels of MDA (Figure 2C).

### 3.7. Effect of AE Extract on Antioxidant Enzymes In Vitro

**Figure 3:**
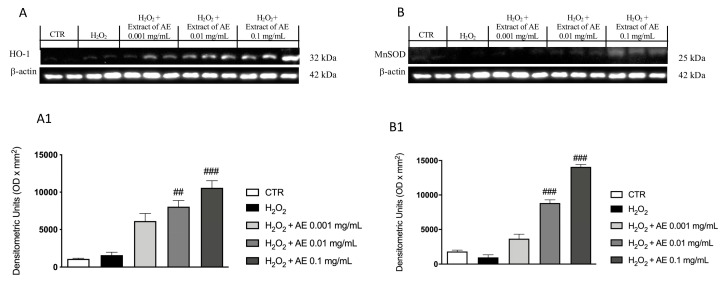
Effect of AE extract on antioxidant enzymes in vitro. Western blot analysis of HO-1 (**A**) and densitometric analysis (**A1**) and MnSOD (**B**) and densitometric analysis (**B1**) showed a significant increase following AE extract pre-treatment in a concentration dependent manner. Data are representative of at least three independent experiments; One-Way ANOVA test. ## *p* < 0.01 vs. H_2_O_2_; ### *p* < 0.001 vs. H_2_O_2_.

The stimulation with H_2_O_2_ caused important oxidative damage that reflected in a changed expression of antioxidant enzymes such as Mn-SOD and HO-1, markers of oxidative stress. Through western blot analysis we evaluated the expression of these enzymes. Our results showed basal levels of both enzymes following H_2_O_2_ stimulation and AE extract at the concentration of 0.01 and 0.1 mg/mL, showing an increase of the expressions of HO-1 (Figure 3A and densitometric analysis Figure 3(A1)) and Mn-SOD (Figure 3B and densitometric analysis Figure 3(B1)), respectively. 

### 3.8. Effect of AE at Different Doses on Paw Edema Volume following CAR-Injection in Rats

**Figure 4:**
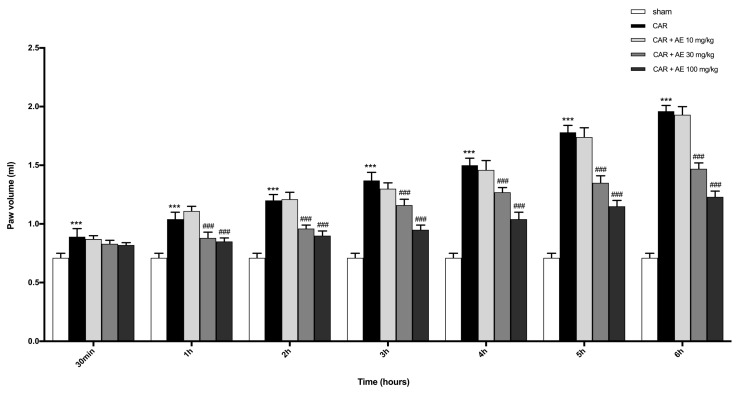
Effect of AE extract on the time-course of CAR-induced paw edema in rat. CAR injection starting from 3h, significantly increased paw volume in a time dependent manner. AE reduced paw volume only at doses of 30 and 100 mg/kg. One-Way ANOVA test. ***p < 0.001 vs. sham; ### *p* < 0.001 vs. CAR.

CAR injected at the subplantar level in the right paw caused a significant increase in the volume of the paw, starting from 2 h post CAR-injection until 6 h (Figure 4). Over the 6 h, a substantial decrease in the volume of the paw edema was detected in rats pre-treated with AE extract at 30 and 100 mg/kg compared to the sham group; while treatment with AE extract at 10 mg/kg did not significantly enhanced paw edema induced by CAR.

### 3.9. Effect of AE Extract on CAR-Induced Histological Damage and Neutrophilic Infiltration

**Figure 5:**
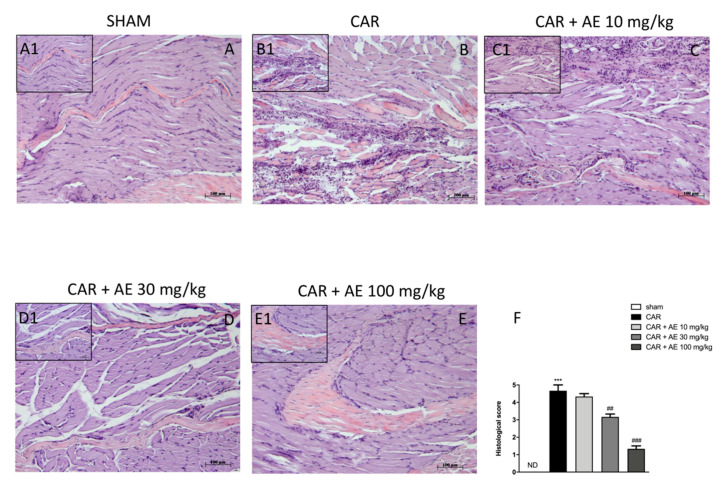
Effect of AE at different doses on paw edema volume following CAR-injection in rats. No histological variations were detected in group 1: Sham + vehicle rats (**A**) and (**A1**), see histological score (**F**). Extensive histological damage was evaluated in group 2: CAR + vehicle rats (**B**) and (**B1**), see histological score (**F**). Group 3: CAR + AE 10 mg/kg has been ineffective in reducing CAR tissue damage (**C**) and (**C1**), see histological score (**F**). CAR tissue damage was reduced by AE + 30 mg/kg (**D**) and (**D1**), see histological score (**F**) and AE + 100 mg/kg (**E**) and (**E1**), see histological score (**F**) treatments. Data are representative of at least three independent experiments; One-Way ANOVA test. *** *p* < 0.001 vs. sham; ## *p* < 0.01 vs. CAR; ### *p* < 0.001 vs. CAR. ND not detectable.

H/E staining revealed the structural integrity of the tissue sections. The tissues from sham rats showed no histological alterations and the muscle fibers maintained its integrity (Figure 5(A,A1), see histological score Figure 5F). The CAR group showed disorganization in the structure of the muscle fibers, with an increase in the space between the fibers (Figure 5(B,B1)), see histological score Figure 5F) and a significant increase in inflammatory cells infiltration, which confirmed the positive course of the inflammatory reaction caused by the induction of edema. Rats with AE extract, at a dose of 10 mg/kg still showed a significant infiltration of inflammatory cells, edema formation, and muscle fiber disorganization (Figure 5(C,C1), see histological score Figure 5F). However, treatment with AE extracts at the dose of 30 and 100 mg/kg significantly improve the normal structure of the fibers and showed an important reduction of inflammatory cells (Figure 5(D,D1) and Figure 5(E,E1) respectively; see histological score Figure 5F) in a dose dependent manner.

**Figure 6:**
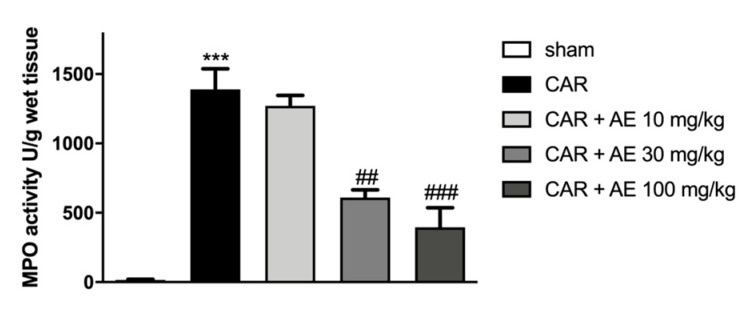
Effect of AE extract on neutrophil infiltration in rat paw tissue. MPO analysis showed basal levels in the sham group, instead, we detected an increase of MPO levels in the CAR group. AE extract, in a dose dependent manner, showed a reduced MPO activity. Data are representative of at least three independent experiments; One-Way ANOVA test. *** *p* < 0.001 vs. sham; ## *p* < 0.01 vs. CAR; ### *p* < 0.001 vs. CAR.

To evaluate the infiltration of inflammatory cells, already revealed by staining with H/E, we measured MPO activity in paw tissues. MPO is a well noted marker of neutrophilic infiltration. Sham-group revealed a basal level of MPO instead an increase in MPO activity was found in rats that received the injection of CAR (Figure 6). The administration of AE extract at 30 mg/kg significantly reduced MPO activity, an activity that was even more reduced by the AE extract at a dose of 100 mg/kg. AE extract at a dose of 10 mg/kg did not produce a reduction in the infiltration of neutrophils into the paw tissues. 

### 3.10. AE Extract Modulated Pro-Inflammatory Enzymes and NF-κB Pathway in Rat Paw Tissue

**Figure 7:**
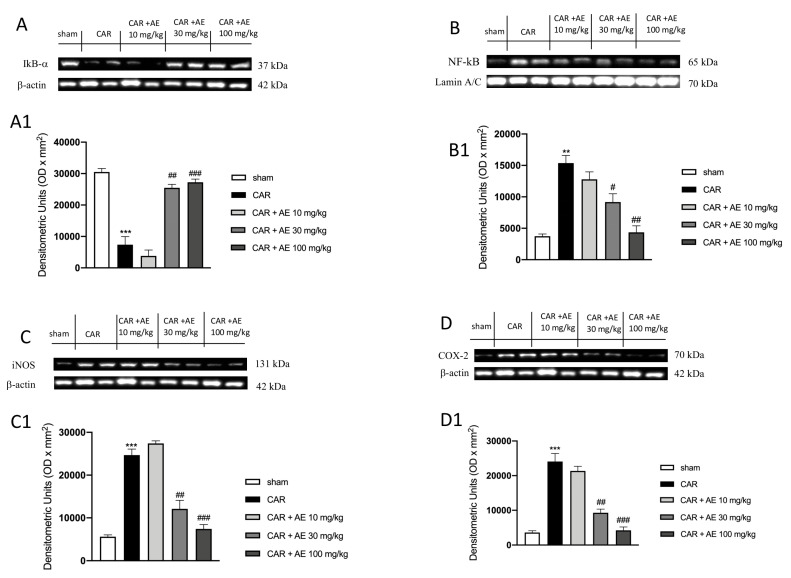
Effect of AE extract on NF-κB pathway and pro-inflammatory enzymes. IκB-α basal levels were identified in sham animals; while an increase in the degradation of IκB-α was found in CAR group. AE 10 mg/kg didn’t increase IκB-α levels; AE 30 mg/kg and 100 mg/kg restored IκB-α expression (**A**) and densitometric analysis (**A1**). NF-κB expression was significantly increased in the CAR group compared to the sham group; such expression was decreased by AE 30 mg/kg and 100 mg/kg treatments but not from 10 mg/kg (**B**) and densitometric analysis (**B1**). Analysis of iNOS (**C**) and densitometric analysis (**C1**) and COX-2 (**D**) and densitometric analysis (**D1**) showed basal level in the sham group that was significantly increased in the CAR group. AE 10 mg/kg did not reduce COX-2 and iNOS expressions; contrarily AE extract at two highest doses, significantly reduced these expressions. Data are representative of at least three independent experiments; One-Way ANOVA test. ** *p* < 0.01 vs. sham; *** *p* < 0.001 vs. sham; # *p* < 0.05 vs. CAR; ## *p* < 0.01 vs. CAR; ### *p* < 0.001 vs. CAR.

To verify the anti-inflammatory activity of the extract of AE, we analyzed through western blot analysis, the modulation of the NF-κB pathway. We found a basal level of IκB-α in the sham group (Figure 7A and densitometric analysis Figure 7(A1)) whereas IκB-α levels were significantly down-regulated in rats injected with CAR (Figure 7A and densitometric analysis Figure 7(A1)). Treatment with AE extract at the dose of 10 mg/kg, didn’t restore the levels of IκB-α compared to the sham group (Figure 7A and densitometric analysis Figure 7(A1)). However, the administration of AE extract at the dose of 30 mg/kg, and more significantly at the dose of 100 mg/kg significantly restored IκB-α levels as sham levels (Figure 7A and densitometric analysis Figure 7(A1)). Moreover, the translocation of NF-κB in the nucleus was significantly increased in the CAR group (Figure 7B and densitometric analysis Figure 7(B1)), compared to the sham group (Figure 7B and densitometric analysis Figure 7(B1)). Treatment with AE extract at a dose of 30 and 100 mg/kg significantly reduced NF-κB levels in a dose dependent manner, (Figure 7B and densitometric analysis Figure 7(B1)), while the administration of AE extract at a dose of 10 mg/kg was ineffective (Figure 7B and densitometric analysis Figure 7(B1)). Translocation of NF-κB into the nucleus determines the production and activation of pro-inflammatory enzymes such as iNOS and COX-2. Thus, by western blot analysis, we evaluated the levels of both enzymes as showed in Figure 7C,D respectively. Western Blot analysis of iNOS and COX-2 showed basal expression in the sham group, an expression that was significantly increased in the CAR group (Figure 7C,D respectively and densitometric analysis Figure 7(C1,D1)). Treatment with AE extract 10 mg/kg did not show a significant reduction in iNOS and COX-2 levels, whereas this expression was significantly reduced by treatment with AE extract at the dose of 30 mg/kg and even more at the dose of 100 mg/kg (Figure 7C,D respectively and densitometric analysis Figure 7(C1,D1)). 

### 3.11. AE Extract Improved the Antioxidant Response in Rat Paw Tissue

**Figure 8:**
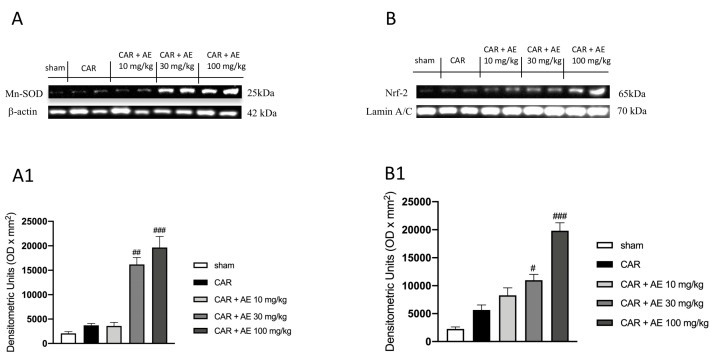
Effect of AE extract on antioxidant response in rat paw issue. Basal levels of MnSOD and Nrf2 were found in sham and CAR groups. AE extract, in a dose dependent manner, promoted the antioxidant response, increasing MnSOD expression (**A**) and densitometric analysis (**A1**) and Nrf2 (**B**) and densitometric analysis (**B1**). Data are representative of at least three independent experiments; One-Way ANOVA test. # *p* < 0.05 vs. CAR; ## *p* < 0.01 vs. CAR; ### *p* < 0.001 vs. CAR.

We also analyzed the expression of Mn-SOD, a mitochondrial antioxidant enzyme that catalyzes the conversion of superoxide radicals to hydrogen peroxide and the expression of Nrf-2 a transcription factor that regulates the gene expression of a large variety of antioxidant cytoprotective enzymes. Our results showed basal levels of both enzymes in the sham group (Figure 8A,B respectively and densitometric analysis Figure 8(A1,B1)), this highlighted a physiological condition in which the animal does not show a powerful antioxidant response as there is no strong oxidative stress condition to be countered. Differently, AE extract at the dose of 30 mg/kg but even more significantly at the dose of 100 mg/kg dose, improved the antioxidant response (Figure 8A,B respectively and densitometric analysis Figure 8(A1,B1)), promoting a better action in countering the damage produced by the induction of edema.

## 4. Discussion

Food supplements constitute a great source of nutrients like vitamins and minerals [42]; most of the dietary supplements on commerce are derived from our ecosystem, and in this wide range, marine resources should not be underestimated [17]. The marine ecosystem contains a large variety of living beings with biological activity that could support research and improve the development of new drugs and food supplements [43,44].

This study evidenced that within AE are detectable polyphenols and phytosterols that probably may be related to phytoplankton origin or to the common presence of macroalgal fragments in AE coelenterons. These data, combined with the results on n-6/n-3 ratio for fatty acids, and the already known high carotenoids content, suggested that the use of AE such as an aliment can be associated to beneficial health effects.

AE has a great variety of biological properties still not fully known [45], and therefore it represents an unexplored source to be studied with great attention. Some in-vitro studies have highlighted some anti-inflammatory effects [46], as well as anticancer activities [47,48], and antimicrobial potential [49]; however, few are the studies have been conducted in vivo and on anti-oxidant properties; therefore, the purpose of this research was to examine biological properties through in-vitro and in-vivo studies. 

Oxidative stress is a condition in which there is an alteration between the production and the elimination of oxidizing chemical species, as H_2_O_2_, and the antioxidant defense systems; this leads to a significant increase in free radicals as reactive oxygen or nitrogen species [50], representing a harmful event for cells and tissues of our body [51]. 

Pre-treatment with AE extract has been shown to have good antioxidant properties, reducing the cytotoxic action of oxidizing agent H_2_O_2_ as confirmed by the test carried out on macrophage cells. Another parameter used to evaluate the antioxidant activity was the MDA level; its increase is related to alterations in the DNA structure and to the presence of peroxide free radicals that favor oxidative stress conditions [52]. Our results showed that AE extract was effective in reducing MDA levels. Antioxidant enzymes play a fundamental role in neutralizing the dangerous action of free radicals, thus exerting a protective action on cellular integrity [53]; the obtained results indicated a significant up-regulation of HO-1 and MnSOD expression following the treatment with AE. All of these data highlighted the ability of AE extract to increase the response against oxidative stress. 

Following the in vitro study, we carried out an in vivo study to evaluate the beneficial properties of AE during acute inflammation. For this purpose, we used a model of paw edema induced by CAR, a substance able to induce inflammation in few hours, which represents an experimental model well validated in the scientific environment [18,20]. The administration of CAR induces a typical acute inflammatory condition of the paw, with the appearance of the classic signs of inflammation, like edema [54], which becomes evident after 3-4 h from inoculation. Our results demonstrated that AE extract was able to inhibit the increase of paw edema due to CAR injection. Acute inflammation, due to CAR injection, caused in the paw a deterioration of muscle fibers and inflammatory cell infiltration [55]. However, the administration of AE extract visibly reduced the histological damage induced by CAR, decreasing the morphological alterations of tissue architecture and the presence of neutrophil infiltrate that was also confirmed by MPO level [56], a local mediator of tissue damage and the resulting inflammation in many inflammatory diseases [48,57]. NF-κB is a protein complex that acts as a transcription factor, which covers a key role in inflammatory processes and many other diseases [58]. The translocation of NF-κB in the nucleus promotes the transcription of specific genes that involve the production of pro-inflammatory cytokines [59]. AE extract, in a dose-dependent manner, showed the ability to modulate the NF-κB pathway, decreasing the degradation of IκB-α and, consequently, the translocation of NF-κB in the nucleus, leading to lower production of pro-inflammatory enzymes such as iNOS and COX-2. The correlation between NF-κB and Nrf-2 signaling pathways, demonstrated in many scientific studies, reveals a cross-talking between the inflammatory state and oxidative stress [59,60]. 

Nrf2 is an important cytoprotective transcription factor that has a fundamental role in cellular homeostasis; it promotes the transcription of various anti-oxidant enzymes that contrast oxidative stress conditions present in the cellular environment [60,61]. 

On this basis, we looked at the Nrf-2 pathway to better understand the role of AE in the management of oxidative stress. Our results showed that AE increased the antioxidant response, upregulating the expressions of Nrf2 and MnSOD, and thus suggesting a promising activity to counteract oxidative stress induced by the edematous condition.

## 5. Conclusions

In conclusion, this study indicated that AE extract has powerful antioxidant and anti-inflammatory activity acted by the modulation of the NF-κB/Nrf-2 pathway. Furthermore, this extract could represent a good starting point for the realization of a food supplement, which could prevent the onset of inflammatory pathology, improving the state of health and well-being, as well as quality of life of the patients.

## Figures and Tables

**Table 1:** Fatty acid composition (% of total fatty acids) of total lipids in *Actinia equina* sample.

Name	Fatty Acids	%
butyric acid	C4:0	0.13
caproic acid	C6:0	0.15
caprylic acid	C8:0	0.03
capric acid	C10:0	0.03
undecanoic acid	C11:0	0.02
lauric acid	C12:0	0.06
myristic acid	C14:0	4.41
pentadecanoic acid	C15:0	1.03
palmitic acid	C16:0	28.53
margaric acid	C17:0	2.56
stearic acid	C18:0	11.26
arachidonic acid	C20:0	0.14
heneicosanoic acid	C21:0	0.53
behenic acid	C22:0	0.11
tricosylic acid	C23:0	0.18
lignoceric acid	C24:0	1.37
	∑ SAFAs	50.54
myristoleic acid	C14:1	0.19
*cis-10*-pentadecenoic acid	C15:1	0.10
palmitoleic acid	C16:1 (n-7)	3.49
*cis-10*-heptadecenoic acid	C17:1 (n-7)	0.32
oleic acid	C18:1 (n-9)	8.51
vaccenic acid	C18:1 (n-7)	7.07
*cis-11*-eicosenic acid	C20:1 (n-9)	2.16
erucic acid	C22:1 (n-9)	0.20
nervonic acid	C24:1 (n-9)	1.22
	∑ MUFA	23.26
linoleic acid	C18:2 (n-6)	2.11
γ-linolenic acid	C18:3 (n-6)	0.09
α-linolenic acid	C18:3 (n-3)	0.99
stearidonic acid	C18:4 (n-3)	n.d.
*cis-11,14*-eicosadienoic acid	C20:2 (n-6)	1.95
*cis-8,11,14*-eicosatrienoic acid	C20:3 (n-6)	2.58
*cis-11,14,17*-eicosatrienoic acid	C20:3 (n-3)	1.18
arachidonic acid	C20:4 (n-6)	2.77
*cis*-*5,8,11,14,17*-eicosapentaenoic acid	C20:5 (n-3)	9.07
*cis-13,16*-docosatetraenoic acid	C22:2 (n-6)	0.30
*cis-4,7,10,13,16,19*-docosahexaenoic acid	C22:6 (n-3)	5.16
	∑ PUFAs	26.20
	∑n-3 PUFAs	16.40
	∑n-6 PUFAs	9.50
	n-6/n-3	0.58
	∑total FAs	100

Saturated fatty acids (SAFAs); polyunsaturated fatty acids (PUFAs).

**Table 2:** Phytosterol and triperpenic alcohol composition in *Actinia equine*. Concentration (mg/kg) of single compounds are expressed as mean ± s.d. (*n* = 3).

Compound	Concentration (mg/kg)	%
cholesterol	22,318.54 ± 67.50	94.96
brassicasterol	1.11 ± 0.05	0
ergosterol	390.93 ± 4.69	1.63
campesterol	227.69 ± 3.87	0.97
campestanol	54.63 ± 0.93	0.22
stigmasterol	n.d. ^1^	0
Δ-7-campesterol	28.97 ± 1.02	0.12
clerosterol	14.04 ± 0.08	0.06
*β*-sitosterol	582.71 ± 2.72	2.48
sitostanol	n.d.	0
Δ-5-avenasterol	264.9	1.13
Δ-5,24-stigmastanol	24.86 ± 0.58	0.11
Δ-7-stigmastenol	69.87 ± 0.89	0.30
Δ-7-avenasterol	33.45 ± 1.01	0.13
**Total area**	24,012	100

^1^ n.d.: not detected.

**Table 3:** Compounds identified *Actinia equina*, with corresponding retention times (Rt), UV-vis spectra data (DAD), APCI(-)/MS data, and relative amounts (mg/kg dry weight). Concentration (mg/kg) of single compounds are expressed as mean ± s.d. (*n* = 3).

ID	Compound	Rt	PDA	APCI(-)/MS	Concentration
		(min)	λ max	(*m/z*)	(mg/kg)
1	astaxanthin	11.60	476, 483, 486	596	255.7 ± 10.2
2	lutein	18.51	445, 473	568	150.3 ± 12.7
3	actnioerythrin	25.63	460, 496, 528	564	260.2 ± 13.8
4	α-carotene	34.40	422, 444, 473	536	344.1 ± 16.5
5	β-carotene	41.23	425, 451, 478	536	550.6 ± 23.5
6	violerythrin	46.88	521, 556, 591	564	1328.6 ± 39.7

**Table 4:** Polyphenols revealed in *Actinia equina* by RP-UHPLC-DAD-MS. Concentration (mg/kg) of single compounds are expressed as mean ± s.d. (*n* = 3).

Compound	Concentration
	(mg/kg)
*p*-coumaric acid	8.03 ± 1.70
catechin	15.32 ± 2.23
quercetin	51.12 ± 5.08
rutin	36.22 ± 3.17
luteolin	2.53 ± 0.64

**Table 5:** Amino acids composition of *Actinia equina* hydrolysates analyzed by NMR.

Compound	Symbol	Measured μM Concentration by NMR	Relative Weight by NMR %
Isoleucine	ILE	3.46 ± 0.07	10.60
Leucine	LEU	2.18 ± 0.06	6.64
Lysine	LYS	1.85 ± 0.05	6.27
Methionine	MET	0.58 ± 0.03	2.03
Phenylalanine	PHE	1.38 ± 0.04	5.30
Threonine	THR	2.00 ± 0.05	5.54
Valine	VAL	2.24 ± 0.06	6.09
Arginine	ARG	1.46 ± 0.05	5.90
Glycine	GLY	4.76 ± 0.08	8.30
Proline	PRO	2.70 ± 0.08	7.20
Tyrosine	TYR	1.27 ± 0.03	5.35
Alanine	ALA	3.49 ± 0.08	7.20
Glutamic acid	GLU	2.66 ± 0.09	9.05
